# Supplementary material for: 9-Methylfascaplysin Is a More Potent Aβ Aggregation Inhibitor than the Marine-Derived Alkaloid, Fascaplysin, and Produces Nanomolar Neuroprotective Effects in SH-SY5Y Cells
Source: Mar Drugs. 2019 Feb 18;17(2):121. doi: 10.3390/md17020121 (PMC6409607; doi:10.3390/md17020121)
Supplement: Supplementary file 1 [file marinedrugs-17-00121-s001.pdf]

marine drugs

Supplementary Materials

9-Methylfascaplysin is a More Potent Aβ Aggregation Inhibitor than the Marine-Derived Alkaloid, Fascaplysin, and Produces Nanomolar Neuroprotective Effects in SH-SY5Y Cells

Qingmei Sun [1,2,†], Fufeng Liu [3,†], Jingcheng Sang [3], Miaoman Lin [4], Jiale Ma [4], Xiao Xiao [1], Sicheng Yan [1], C. Benjamin Naman [2], Ning Wang [2], Shan He [2], Xiaojun Yan [2], Wei Cui [1,2,3,*] and Hongze Liang [4,*]

[1] Ningbo Key Laboratory of Behavioral Neuroscience, Zhejiang Provincial Key Laboratory of Pathophysiology, School of Medicine, Ningbo University, Ningbo 315211, China; 15658223676@163.com (Q.S.); xx15058492711@163.com (X.X.); Yansicheng9@163.com (S.Y.)
[2] Li Dak Sum Yip Yio Chin Kenneth Li Marine Biopharmaceutical Research Center, College of Food and Pharmaceutical Sciences, Ningbo University, Ningbo 315211, China; bnaman@nbu.edu.cn (C.B.N.); wangning2@nbu.edu.cn (N.W.); heshan@nbu.edu.cn (S.H.); yanxiaojun@nbu.edu.cn (X.Y.)
[3] Key Laboratory of Industrial Fermentation Microbiology of Education, State Key Laboratory of Food Nutrition and Safety, College of Biotechnology, Tianjin University of Science & Technology, Tianjin 300457, China; fufengliu@tust.edu.cn (F.L.); bixian2b@163.com (J.S.)
[4] School of Materials Science and Chemical Engineering, Ningbo University, Ningbo 315211, China; lmm_dll@163.com (M.L.), mjl2137297289@163.com (J.M.)
[5] Key Laboratory of Anesthesiology of Zhejiang Province, The second Affiliated Hospital and Yuying Children's Hospital of Wenzhou Medical University Wenzhou 325035 China; wangdizhu007@163.com (H.W.)
* Correspondence: cuiwei@nbu.edu.cn (W.C.); lianghongze@nbu.edu.cn (H.L.)
† These authors contribute equally

The synthesis of fascaplysin (**3a)** and 9-methylfascaplysin (**3b**). All chemicals were purchased in analytical grade and used as received. Nuclear magnetic resonance spectra of ^{1}H NMR and ^{13}C NMR were recorded on a Bruker AV-400 NMR spectrometer in CD_3OD or DMSO-d_6. HRMS was performed on a Bruker micrOTOF-Q II mass spectrometer or on an Orbitrap Mass Spectrometer (Q Exactive HF, Thermo Fisher Scientific).

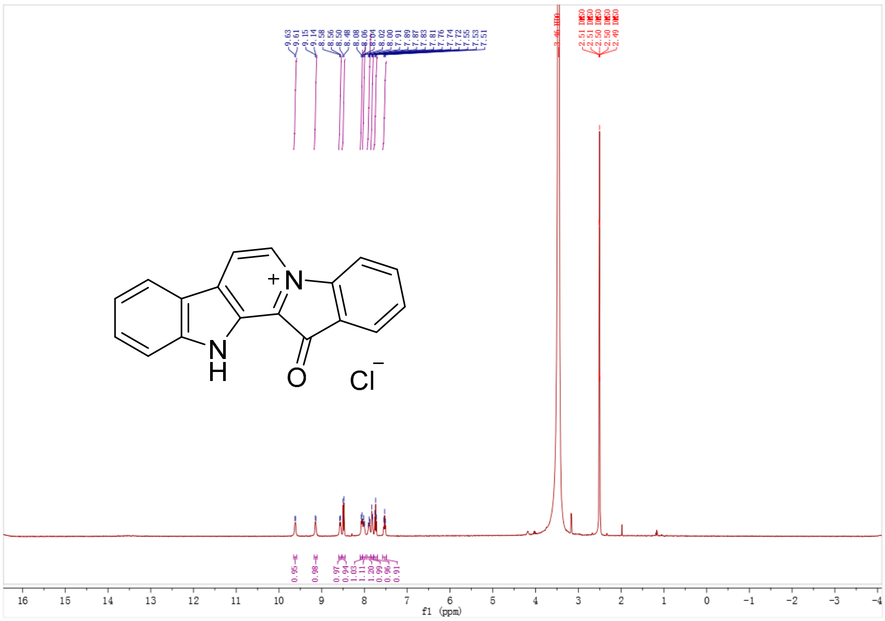

Figure S1. NMR of 3a.

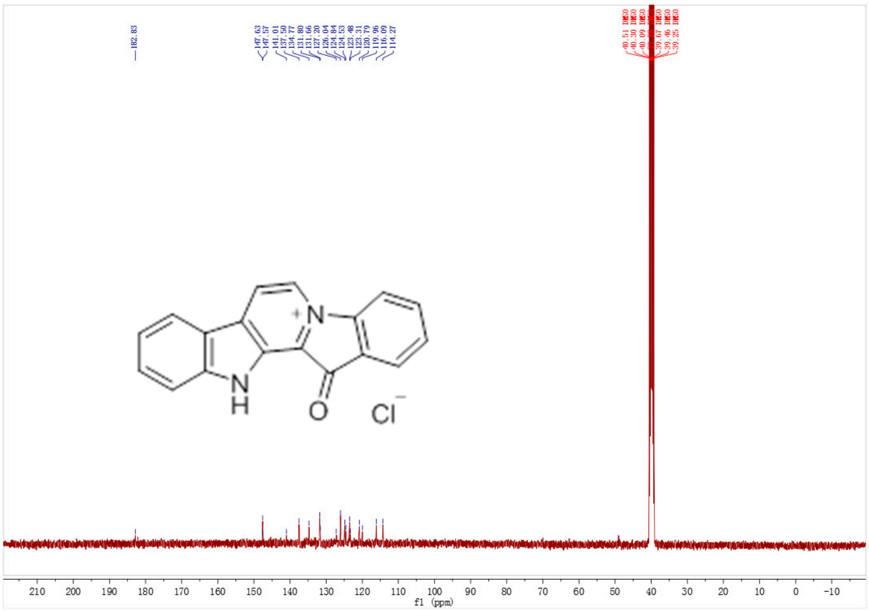

Figure S2. NMR of 3a.

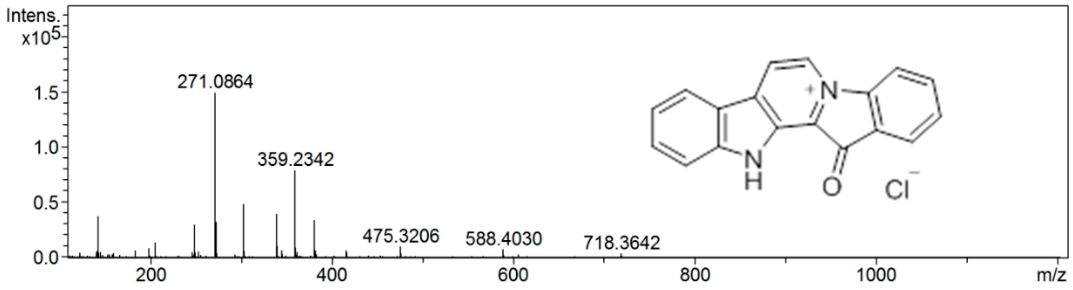

Figure S3. HRMS of 3a.

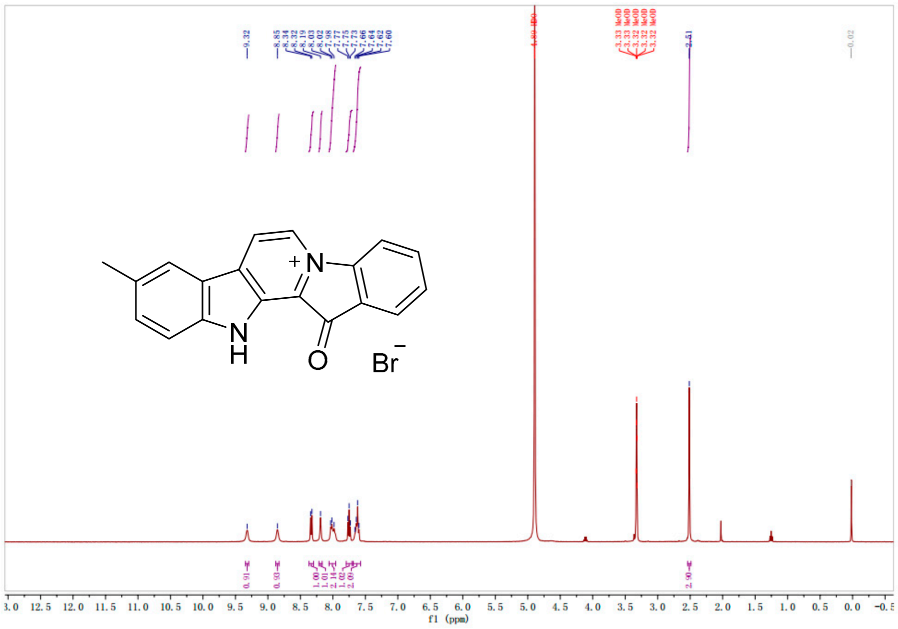

Figure S4. NMR of **3b** (in CD_3OD).

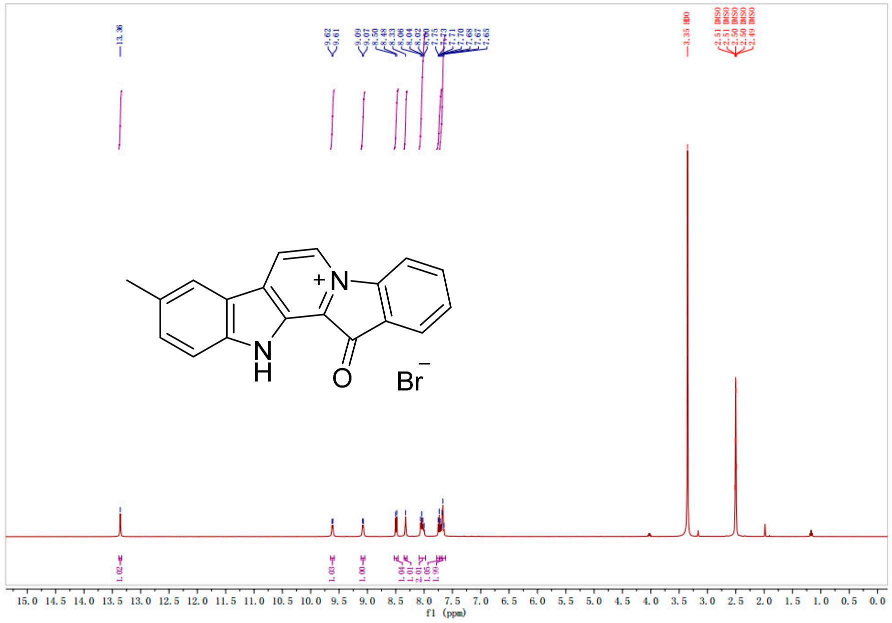

Figure S5. NMR of **3b** (in DMSO-d_6).

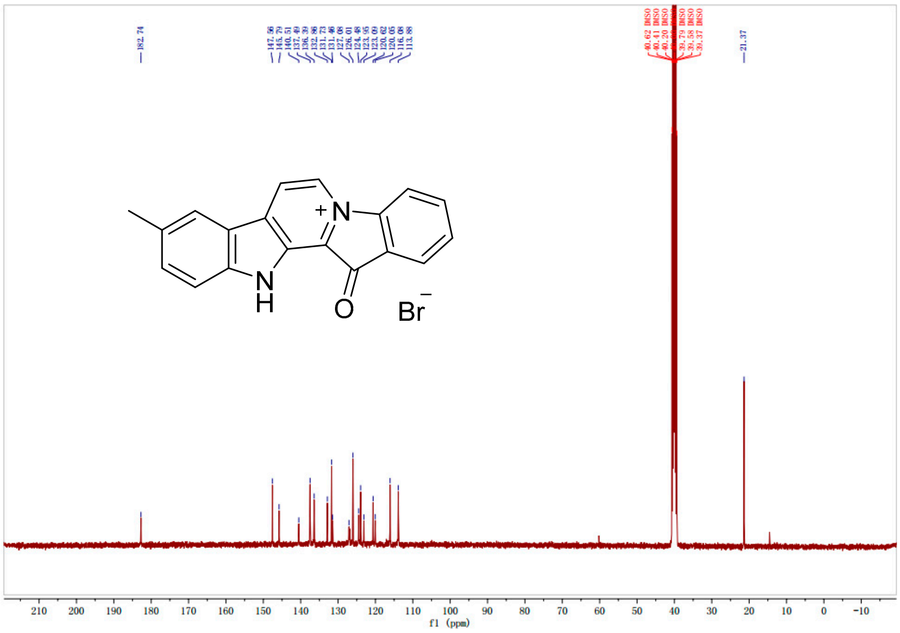

Figure S6. NMR of **3b** (in DMSO-d6).

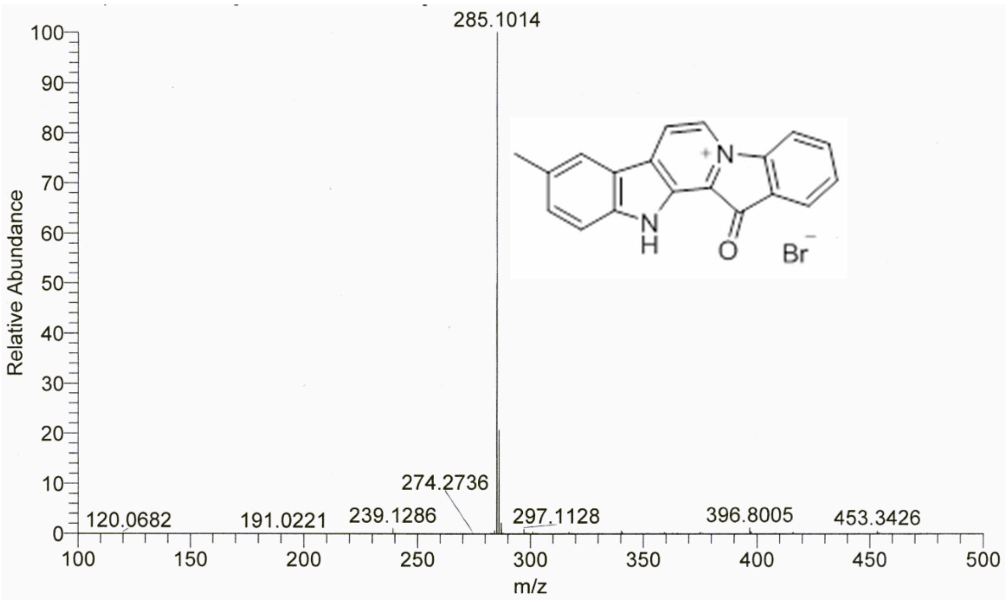

Figure S7. HRMS of 3b.

Measurement information:
Instrument: CXTH LC-3000
Column: Ultimate® Plus C18 (Welch)
Mobile phase: Methanol/water (90/10, V/V)
Velocity: 1 mL/min
Monitor wave number: 280 nm
Temperature: 25 °C
Injection volume: 20 μL

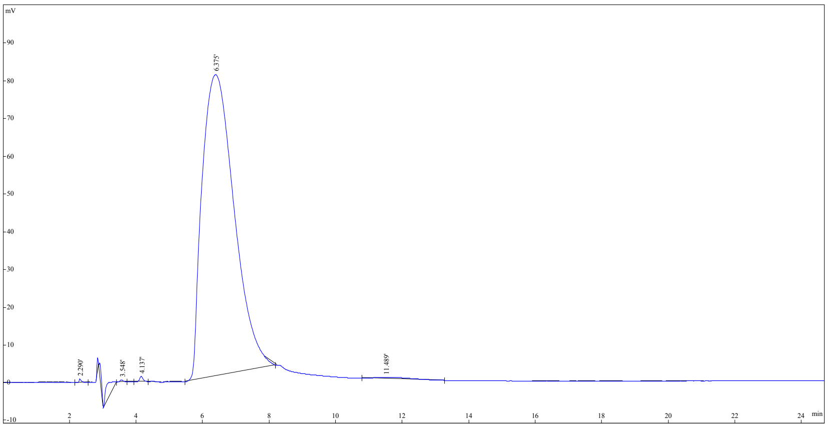

Figure S8. HPLC of 3a.

Peak	Ret. Time	Area (%)	Area
1	2.290	0.1042	5641
2	3.548	0.07692	4164
3	4.137	0.2029	40984
4	6.375	98.96	5357259
5	11.489	0.6564	35537
Total		100	5413585

Measurement information:
Instrument: CXTH LC-3000
Column: Ultimate® Plus C18 (Welch)
Mobile phase: Methanol/water (90/10, V/V)
Velocity: 1 mL/min
Monitor wave number: 280 nm
Temperature: 25°C
Injection volume: 20 μL

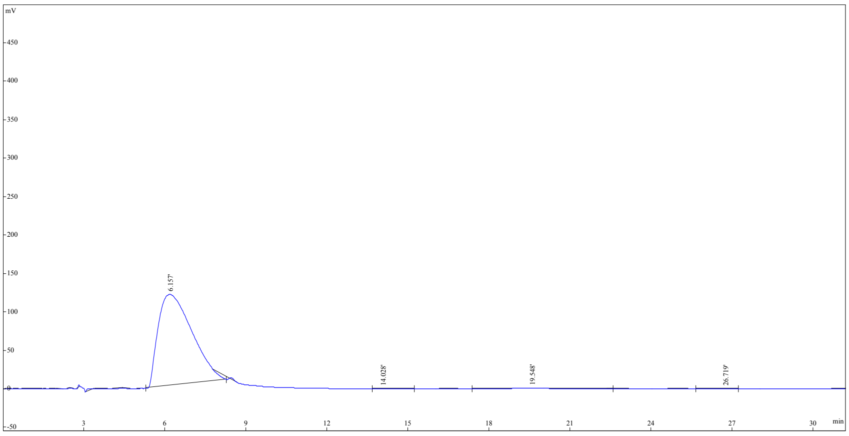

Figure S9. of 3b.

Peak	Ret. Time	Area (%)	Area
1	6.157	98.96	10346263
2	14.028	0.04309	4506
3	19.548	0.9536	99703
4	26.719	0.05022	5251
Total		100	10455723
